# Diaphragmatic breathing during virtual reality exposure therapy for aviophobia: functional coping strategy or avoidance behavior? a pilot study

**DOI:** 10.1186/s12888-016-1181-2

**Published:** 2017-01-18

**Authors:** Youssef Shiban, Julia Diemer, Jana Müller, Johanna Brütting-Schick, Paul Pauli, Andreas Mühlberger

**Affiliations:** 1 Department of Psychology (Clinical Psychology and Psychotherapy), University of Regensburg, Universitätsstraße 31, 93053 Regensburg, Germany; 2 Department of Psychology (Biological Psychology, Clinical Psychology and Psychotherapy), University of Würzburg, Marcusstraße 9-11, 97070 Würzburg, Germany

**Keywords:** Virtual reality, Exposure therapy, Diaphragmatic breathing, Aviophobia, Experimental study

## Abstract

**Background:**

Although there is solid evidence for the efficacy of in vivo and virtual reality (VR) exposure therapy for a specific phobia, there is a significant debate over whether techniques promoting distraction or relaxation have impairing or enhancing effects on treatment outcome. In the present pilot study, we investigated the effect of diaphragmatic breathing (DB) as a relaxation technique during VR exposure treatment.

**Method:**

Twenty-nine patients with aviophobia were randomly assigned to VR exposure treatment either with or without diaphragmatic breathing (six cycles per minute). Subjective fear ratings, heart rate and skin conductance were assessed as indicators of fear during both the exposure and the test session one week later.

**Results:**

The group that experienced VR exposure combined with diaphragmatic breathing showed a higher tendency to effectively overcome the fear of flying. Psychophysiological measures of fear decreased and self-efficacy increased in both groups with no significant difference between the groups.

**Conclusions:**

Our findings indicate that diaphragmatic breathing during VR exposure does not interfere with the treatment outcome and may even enhance treatment effects of VR exposure therapy for aviophobic patients.

**Trial registration:**

Retrospectively registered. ClinicalTrials.gov NCT02990208. Registered 07 December 2016.

## Background

Up to 40% of the population in industrialized countries suffers from a fear of flying; another 20% experiences strong fear during flying [1, 2]. In order to control their fear, many use coping strategies that include the avoidance of fear-related situations, self-medication or alcohol [3]. Also, in clinical practice, various coping strategies based on distraction are commonly used in order to help patients deal with anxiety-inducing situations [4, 5]. However, there is a debate amongst researchers whether such “coping strategies” aiming to reduce fear during treatment are beneficial for the treatment effect.

For example, avoidance of fear-inducing situations or trying to distract oneself is considered, by some authors, to be an adaptive technique for anxious people reacting to threatening situations [6, 7]. Others hold the opinion that short-term relief of anxiety by distraction or avoidance is counterproductive and interferes with effective treatment, therefore maintaining anxiety in the long term [8, 9]. The results by Oliver and Page [10] support the assumption that distraction is an appropriate coping style by showing a beneficial effect of distraction techniques during in vivo exposure treatment for participants with a fear of blood, injections, and/or injury. The participants also displayed an additional increase of subjective control over their anxiety during the month following the exposure session. Johnstone and Page [11] found that spider phobic patients who used distraction during treatment showed higher self-efficacy and subjective control ratings as well as better performance on behavioral tasks after the exposure treatment. However, as far as the physiological reaction during distracted exposure is concerned, the authors reported no difference in blood pressure, heart rate and skin conductance level between the distracted group and the focused group. In another study, Kamphuis and Telch [12] examined participants with claustrophobic fears and found that participants who focused on fear stimuli in the exposure later demonstrated a lower reoccurrence of fear. Although the distraction caused an attenuation of fear during treatment, it did not influence treatment outcome measures (for a review see [13]).

Available evidence about the effects distraction on exposure treatment is inconclusive. In their literature review, Parrish et al. [4] come to the conclusion that strategies for controlling one’s anxiety can be beneficial only if it helps to increase self-efficacy and supports therapeutic change, without diverting attention from the anxiety-related stimuli during exposure therapy. It is important to note that in a review by Craske et al. [13], the authors have not yet found evidence for a relationship between self-efficacy and treatment effects following exposure therapy. However, most studies examined used momentary fear levels during treatment as a dependent variable and only one used a follow-up test. A recent meta-analysis by Fentz, Arendt, O'Toole, Hoffart, and Hougaard [14] also found no studies showing a relationship between *panic self-efficacy* and treatment effects on a follow-up test.

In the present study, we aimed to investigate the role of relaxation as a coping strategy that could increase self-efficacy during treatment for a fear of flying. Relaxation was induced in this study through instructed diaphragmatic breathing (DB) during treatment. DB may be beneficial for exposure therapy since it reduces arousal on the physiological level [15] but at the same time does not divert attention from the feared situation to the same extent as other coping strategies. A few studies have already implemented DB as a coping strategy with mixed results. Biggs, Kelly, and Toney [16] compared the effectiveness of DB vs. focused attention as a coping strategy in dental phobia. The authors failed to demonstrate significant differences between the groups, but found an overall trend in anxiety reduction for all groups. A related study that investigated the effects of biofeedback in aviophobia therapy was conducted by Wiederhold et al. [17]. In this study, both groups were given instructions on how to use DB and the experimental group received an additional biofeedback during treatment. The authors showed that biofeedback had a positive effect on treatment outcome.

For the treatment protocol, we chose Virtual Reality Exposure Treatment (VRET) as it is easily controlled during the exposure and therefore optimal for assessing various process aspects of exposure therapy. In general, it has proven effective for the treatment of anxiety (for a review see the meta-analysis of [18]). Importantly, former studies have demonstrated the efficacy of our treatment protocol for a fear of flying [19] even after a single treatment session [2, 20], as well as the efficacy for VRET treatment protocols of other researcher groups ([21]; [22]). Furthermore, VRET is an economic and safe alternative to in vivo or imagined exposure when examining fear of flying in participants [23–25].

The present study, designed as an inferiority trial, investigated the effect of diaphragmatic breathing as an additional coping strategy during VRET in patients with aviophobia. We assumed that DB would lead to less fear and physiological arousal during the VRET and to an enhanced treatment outcome. In our study, patients with aviophobia received treatment in VR with or without DB. Breathing techniques are often used in fear of flying courses, e.g. in Lufthansa’s seminars for relaxed flying [26]. We assumed that adding DB to VRET would enhance treatment effects by reducing fear during exposure, thus improving the processing of the feared situation. As a result, self-efficacy should be increased in comparison to VRET alone.

## Methods

### Aim

The aim of the study was to investigate the role of a relaxation technique, diaphragmatic breathing, as a coping mechanism during VR exposure therapy of aviophobia.

### Participants

From fifty-nine volunteers who had been recruited via advertisements displayed in public, only thirty (50% male) fulfilled the inclusion criteria (age 20–65, flying experience, subjective rating of fear of flying > 60 from 100). Exclusion criteria were pregnancy, heart disease and current involvement in psychotherapy and/or pharmacotherapy. One participant in the VRET group did not complete the treatment and was excluded from the statistical analyses.

Socio-demographic variables and history of flight behavior are provided in Table 1. There were no pre-treatment differences between groups in reference to socio-demographic characteristics or clinical variables (except for age). On average, participants in the VRET group were about 10 years older than the participants of the VRET + DB group (see Tables 1 and 2).

**Table 1:** Demographic Variables and Diagnoses according to SCID-1

	VRET + breathing		VRET				
Demographics	M	SD	M	SD	df	t	p
Age	34.3	9.81	43	9.96	27	−2.38	*.03*
	**N**	**%**	**N**	**%**			***p*** ^**a**^
Gender							
Female	14	93.3%	10	71.4%			
Male	1	6.7%	4	28.6%			.17
Diagnoses							
Aviophobia	14	93.3%	13	92.9%			1.0
Other specific phobia	9	60.0%	7	50.0%			.72
Panic Disorder	2	13.3%	1	7.1%			1.0
Obsessions	0	--	1	7.1%			.48

Note. Means, Standard Deviations, df-, t- and *p*-Values and also quantity and percentage are given. *M* mean; *SD* standard deviation; *d* degrees of freedom; *VRET* Virtual reality exposure therapy; ^a^Fisher’s exact test, two-tailed

**Table 2:** Independent t – tests for baseline scores

	VRET + breathing	VRET			
Questionnaires	M (SD)	M (SD)	df	t	*p*
ASI (0–4)	1.35 (.43)	1.28 (.62)	27	0.32	.75
FFS (0–4)	2.65 (.45)	2.35 (.78)	27	1.25	.23
Time spent reading the information booklet (min.)	45.7 (20.1)	37.9 (27.0)	27	0.89	.33

Note. Means, Standard Deviations in brackets and also df-, t- and *p*-Values are given. *M* mean; *SD* standard deviation; *df* degrees of freedom; *VRET* Virtual reality exposure therapy; *ASI* Anxiety Sensitivity Index*; FFS* Fear of Flying Scale. *T* - values show the comparison of means between the VRET + breathing group and the VRET group before exposure

In each of the groups, one participant did not fulfill all the criteria for aviophobia (see Table 1). Specifically, these participants did not feel restricted by their fear in their daily lives. They were still included in the study as all other criteria of the specific phobia (flight) were satisfied. The Ethics Committee of the University of Würzburg approved the experiment.

### Apparatus

The VR environment simulated a passenger compartment of a Boeing 737. Motion simulation was achieved with the help of a chair on a 6° of freedom motion platform (Micro-Motion-System, hydraulic, Krauss–Maffei Wegmann GmbH & Co, Munich, Germany), a Head Mounted Display (HMD; Virtual Research HMD V6, Aptos, USA), and a personal computer. A tracking sensor (Fastrak, Polhemus, Vermont, USA) assessed the head movements and updated the field of vision of the HMD. Airplane sounds and security and breathing instructions were provided binaurally through headphones (Sennheiser HD-215, Sennheiser electronic GmbH, Germany). The HMD refreshing rate was 60 Hz, the VR content and tracking latencies were in the millisecond range (<5 ms) [27].

### Psychometric Measures

The *Flying phobia screening questionnaire* (German version FSB [28]) consists of four subscales measuring possible exclusion criteria, etiology, previous coping strategies, intensity of fear of flying and avoidance of flying. The reported retest-reliability for a period of two weeks with *N* = 43 is r_tt_ = .69 [28].

The *Structured Clinical Interview for DSM-IV Disorders* (German version SKID [29]) consists of a brief exploration phase, assessing previous and present symptoms of the patient followed by a structured interview, in which ten sections are processed, each concerning a different psychological disorder based on diagnostic criteria by the Diagnostic and Statistical Manual for mental disorders IV [30].

The *Fear of Flying Scale* (FFS; German version FFB [28]) covers 21 flight situations (e.g., planning the trip, boarding a plane, turbulence during the flight) rated on a 5-point Likert scale. Retest-reliability for the period of three months for the German translation of the FFS is .90 (*N* = 120) and a Cronbach’s α of .83 (*N* = 37) [28]. An unpublished study confirmed these findings: Cronbach’s α = .98 (*N* = 257) and r_tt_ = .87 (*N* = 43). All these scores are close to the original FFS indexes of .94 and .86 [31].

The *Anxiety Sensitivity Index* (ASI [32]) consists of 16 items describing fearful cognitions about anxiety symptoms rated on a five-point scale. The ASI has a retest reliability between *r* = .71 and *r* = .75 [32]. The ASI was administered to test pre-treatment differences between the groups (see Table 2).

*Self–efficacy* was measured based on two questions about air travel (question 1: “How confident are you about flying in a real plane for at least 3 h?” and question 2:” How confident are you that you would experience only a moderate level of fear if you travel by plane?”) and was rated on a 10-point Likert scale (0 = not confident at all, 10 = extremely confident). The questions were administered pre and post VR flights in each of the two sessions.

#### Fear ratings

During the VR flights, patients were asked to rate their current fear on a scale from 0 (no fear) to 100 (extreme fear) at four different moments (in the middle of each of the two turbulence phases and each of the two calm phases).

*Heart rate* (HR) and *skin conductance level* (SCL) were continuously recorded during the VR flights using a V-Amp16 amplifier and the Brain Vision Recorder software (Brain Products GmbH, Gilching, Germany; please see Shiban et al. [33] for details on the analysis and recording).

*Respiration rate* (RR) was measured with the help of a flexible strap placed around the waist. The RR was recorded only in the breathing group with the same setup.

### Experimental design

Participants were randomly assigned to one of the two treatment groups: VRET or VRET + DB. Both groups received information booklets on aviophobia and exposure therapy before the beginning of the exposure session. The VRET group was informed about the importance of exposure without distraction. The VRET + DB group received information on diaphragmatic breathing as a coping strategy during the exposure. One week after the exposure session, a test session consisting of two flights took place. A follow up measurement was implemented after an interval of one year.

### Exposure Session

The first meeting lasted 180 min and consisted of the counseling interview (30 min.) and the exposure session (three VR flights, 75 min.). The second part of the counseling interview focused on the treatment rationale and was different for each group.

### VRET group

In accordance with the standard procedure for exposure therapy, participants were asked to focus on their most anxious thought and/or sensation during each exposure.

### VRET + DB group

The relationship between the respiration rate and emotions as well as its relevance for anxiety management was explained. Diaphragmatic breathing was presented as a treatment extension that allows fearful patients to gain control in situations perceived as emotionally uncontrollable. Patients were trained in the technique of diaphragmatic breathing. They were instructed to take a breath by contracting the diaphragm and were trained to maintain their respiration frequency. They were told to inhale through the nose for four seconds and exhale through the mouth for six seconds (six cycles per minute). Patients then had five minutes to practice by following verbal breathing instructions provided over headphones.

VR exposure was identical in the VRET group, except that the VRET + DB group received breathing instructions over headphones. Each of the three VR flights in the exposure session lasted 22.5 min. and consisted of the following phases: start (8 min.) – calm phase 1 (2 min.) – turbulent phase 1 (2 min.) – calm phase 2 (2 min.) – turbulent phase 2 (2 min.) – landing (6.5 min.).

### Test in VR

The test session took place one week after the exposure session and consisted of two VR flights. The passenger compartment differed from the one in the exposure session in color and, instead of audio security instructions, instructions now appeared on video. This extended the duration of the flights by three minutes. Importantly, no recommendations on breathing were provided.

Each of the VR test flights lasted 25.5 min. and was identical to the exposure sessions in terms of the order and duration of the calm and turbulent flight phases.

### Procedure

Before the exposure session (pre exposure), patients received ASI and FFS questionnaires to be filled out at home. Participants also received the booklet (available from the authors). After the participants gave their written informed consent, the Structured Clinical Interview for DSM-IV Disorders (German version SKID; [29]) was conducted.

Patients completed the Self – Efficacy questionnaire. Then, the electrodes for HR and SCL measurement were attached. Additionally, a strap for measuring the respiratory rate was used (only in the VRET + DB group). Patients were seated on the motion platform and HMD and the headphones were adjusted. Participants again received either the instruction to focus on the fear (VRET) or the advice to concentrate on the breathing process (VRET + DB). Then, the exposure flights took place (separated by a 5-min break). After the third VR flight, patients completed the post exposure questionnaires (FFS, Self-Efficacy). Patients in the VRET + DB group were requested to practice diaphragmatic breathing as often as possible until the next session.

One week later, a test session took place in the same laboratory. Before the session started (pre test), the FFS and Self-Efficacy questionnaires were filled out. In the VRET + DB group, the time spent practicing diaphragmatic breathing at home was noted. Electrodes were attached and patients were seated on the motion platform without any further instructions. During the test, two VR flights took place with a 5-min break between them. Afterwards, the post test questionnaires (FFS, Self-Efficacy) were filled in. After an interval of one year, a postal follow up measurement was implemented using the same questionnaires (FFS, Self-Efficacy) as those in the post test session.

### Statistical analyses

To test for baseline differences between treatment groups, *t*-tests for independent samples were conducted. Outcome measures (mean FFS scores, Self-Efficacy scores) were analyzed in separate ANOVAs with the between-subject factor “group” (VRET + DB, VRET) and the within-subject factor “time” (pre exposure, post exposure, pre test, post test and follow up). The process measures (Fear ratings, HR, SCL) were analyzed using mixed ANOVAs with a repeated-measures design with the between-subject factor “group” (VRET + DB, VRET) and the within-subject factors “flight” (exposure: flight 1, flight 2, flight 3) and “phase” (calm 1, turbulent 1, calm 2, turbulent 2). The significance level was set at α = .05. Significant effects in the ANOVAs were followed up by *t*-tests. Greenhouse-Geisser correction was administered when sphericity assumption was violated.

## Results

### Outcome measures

#### Fear of Flying

**Fig. 1:**
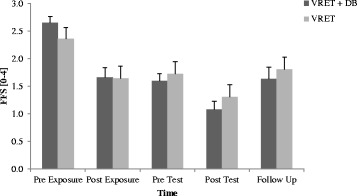
Questionnaire ratings of the Fear of Flying Scale (FFS). Note. VRET = Virtual reality exposure therapy, DB = diaphragmatic breathing. Graphs represent the mean of the questionnaire ratings of the FFS for the five measurements for both groups (VRET + DB: N = 15, VRET: *N* = 14): pre exposure, post exposure, pre test (one week after exposure session), post test and follow up (after one year, VRET + DB: *N* = 12, VRET: *N* = 13). Standard errors are represented as error bars

As we can see in Fig. 1, there was a drop in the participants’ fear of flying in the course of the exposure and test sessions, which was supported by a significant main effect of time (F_2.80, 64.39_ = 38.17, *p* ≤ .001, *η*_*p*_^*2*^ = .62). Furthermore, a marginally significant interaction Time x Group (*F*_2.80, 64.39_ = 2.70, *p* = .057, *η*_*p*_^*2*^ = .11) was evident. Follow-up *t*-tests of the within-subjects factor time revealed a significantly greater decrease in fear of flying from pre exposure [*M* = 2.47, *SD* = 0.68] to the rest of the measurements (post exposure [*M* = 1.59, *SD* = 0.78], pre test [*M* = 1.59, *SD* = 0.72], post test [*M* = 1.09, *SD* = 0.72], and follow up [*M* = 1.72, *SD* = 0.76] all *p*’s < .001).

Excluding two participants older 55 years of age, there was still a significant main effect of time *(F*_2.74, 57.47_ = 32.93, *p* ≤ .001, *η*_*p*_^*2*^ = .61). Furthermore, a significant interaction Time x Group (*F*_2.74, 57.47_ = 3.83, *p* = .017, *η*_*p*_^*2*^ = .15) was evident. Follow-up *t*-tests of the within-subjects factor time revealed a significantly greater decrease of fear of flying from pre exposure [*M* = 2.52, *SD* = 0.67] to the rest of the measurements (post exposure [*M* = 1.70, *SD* = 0.71], pre test [*M* = 1.69, *SD* = 0.66], post test [*M* = 1.17, *SD* = 0.68], and follow up [*M* = 1.77, *SD* = 0.75] all *p*’s < .001).

#### Self-efficacy

**Fig. 2:**
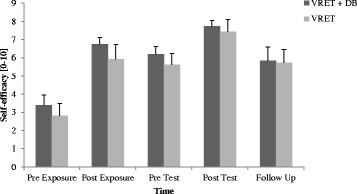
Self-efficacy scores. Note. VRET = Virtual reality exposure therapy, *DB* = diaphragmatic breathing. Graphs represent the mean of the self-efficacy scores for the five measurements for both groups (*VRET + DB: N* = 15, *VRET: N* = 14): pre exposure, post exposure, pre test (one week after exposure session), post test and follow up (after one year, *VRET + DB: N* = 12, *VRET: N* = 14). Standard errors are represented as error bars

As shown in Fig. 2, there was an increase in self-efficacy over time in both groups, which corresponds to a significant main effect of time (*F*_2.67, 64.13_ = 25.26, *p* < .001, *η*_*p*_^*2*^ = .51). No effect involving the factor group was significant.

Excluding the two participants aged above 55 there was a significant main effect of time as well (*F*_2.55, 56.04_ = 23, 07, *p* < .001, *η*_*p*_^*2*^ = .51). No effect involving the factor group was significant.

### Process analysis during exposure session

#### Self-reported fear

**Fig. 3:**
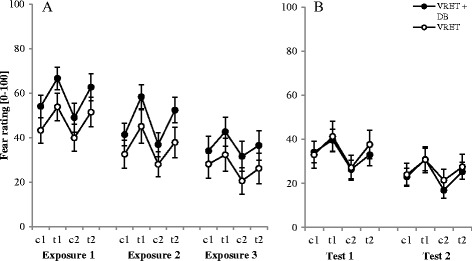
Fear ratings during the exposure (3**a**) and test session (3**b**). Note. VRET = Virtual reality exposure therapy, *DB* = diaphragmatic breathing, *c1/c2* = calm, *t1/t2* = turbulence. Graphs represent the mean of the fear ratings during the exposures (1–3) and the test sessions (1–2) for both groups. Standard errors are represented as error bars

Figure 3 shows that fear ratings changed within flights (depending on the flight phase) and between flights. These changes are indicated by significant main effects of flight (*F*_1.20, 32.26_ = 26.31, *p* = < .001, *η*_*p*_^*2*^ = .49), phase (*F*_1.85, 49.95_ = 23.26, *p* < .001, *η*_*p*_^*2*^ = .46), and a significant Flight x Phase interaction (*F*_3.98, 107.48_ = 4.52, *p* < .001 *η*_*p*_^*2*^ = .14). Group differences were not significant. To further analyze the Flight x Phase interaction, we conducted three separate repeated measures ANOVAs for the three flights. These ANOVAs revealed a significant main effect of phase in all flights (first: *F*_2.40, 67.06_ = 13.67, *p* < .001, *η*_*p*_^*2*^ = .33; second: *F*_1.63, 45.62_ = 25.70, *p* < .001, *η*_*p*_^*2*^ = .48; third: *F*_2.36, 66.04_ = 12.90, *p* < .001, *η*_*p*_^*2*^ = .32). Further *t*-tests for the first flight showed that all pairs were significant (*p* < .05), except two pairs (c1/c2: *p* = .160, t1/t2: *p* = .120). In the second flight, all pairs reached significance (*p* < .05) and in the third flight all except c1/t2 (*p* = .861) did as well.

Excluding the two old participants, there were significant main effects of flight (*F*_1.22, 30.58_ = 20.84, *p* = < .001, *η*_*p*_^*2*^ = .46), phase (*F*_1.73, 43.13_ = 23.40, *p* < .001, *η*_*p*_^*2*^ = .48), and a significant Flight x Phase interaction (*F*_6, 150_ = 4.24, *p* = .001, *η*_*p*_^*2*^ = .15). Group differences were not significant. Repeated measures ANOVAs for the three flights revealed a significant main effect of phase in all flights (first: *F*_2.26, 58.82_ = 16.99, *p* < .001, *η*_*p*_^*2*^ = .39; second: *F*_1.56, 40.62_ = 23.51, *p* < .001, *η*_*p*_^*2*^ = .48; third: *F*_2.37, 61.53_ = 12.82, *p* < .001, *η*_*p*_^*2*^ = .33).

Further *t*-tests for the first flight showed that all pairs were significant (*p* < .05). In the second flight, all pairs reached significance as well (*p* < .05) and in the third flight all except c1/t2 (*p* = .862).

#### Heart rate (HR)

**Fig. 4:**
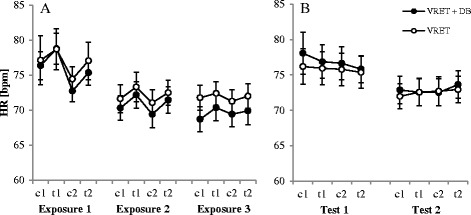
Heart rate (HR) during the exposure (4**a**) and test session (4**b**). Note. VRET = Virtual reality exposure therapy, *DB* = diaphragmatic breathing, *c1/c2* = calm, *t1/t2* = turbulence. Graphs represent the mean of the heart rate during the exposures (1–3) and the test sessions (1–2) for both groups. Standard errors are represented as error bars

As demonstrated in Fig. 4, HR decreased both within and between flights. These patterns were partially confirmed by the statistical analysis. ANOVA revealed significant main effects of the factor flight (*F*_1.41, 38.05_ = 17.13, *p* < .001, *η*_*p*_^*2*^ = .39), phase (*F*_2.13, 57.42_ = 14.41, *p* < .001, *η*_*p*_^*2*^ = .35), and a Flight x Phase interaction (*F*_2.57, 69.26_ = 4.95, *p* < .001, *η*_*p*_^*2*^ = .16). No other effects were significant. We conducted three separate repeated measures ANOVAs for the three flights to follow up on the interaction. We conducted three separate repeated measures ANOVAs for the three flights to follow up on the interaction. These ANOVAs revealed a significant main effect of phase in all three flights (first: *F*_2.04, 57.08_ = 9.52, *p* < .001, *η*_*p*_^*2*^ = .25; second: *F*_2.22, 62.13_ = 13.65, *p* < .001, *η*_*p*_^*2*^ = .33; third: *F*_2.31, 64.73_ = 4.60, *p* = .010, *η*_*p*_^*2*^ = .14). Further *t*-tests for the first flight showed that all pairs were significant (*p* < .05), except the pair (c1/t2: *p* = .512). In the second flight, all pairs reached significance (*p* < .05), except one pair that was marginally significant (c1/c2: *p* = .057). In the third flight, only 4 out of 6 pairs were significant (*p* < .05; c1/c2: *p* = .686, t1/t2: *p* = .237).

Excluding the two older participants, an ANOVA revealed significant main effects of the factor flight (*F*_1.41, 35.30_ = 15.92, *p* < .001, *η*_*p*_^*2*^ = .39), phase (*F*_2.09, 52.19_ = 13.32, *p* < .001, *η*_*p*_^*2*^ = .35), and a Flight x Phase interaction (*F*_2.46, 61.50_ = 5.06, *p* < .001, *η*_*p*_^*2*^ = .17). No other effects were significant. We conducted three separate repeated measures ANOVAs for the three flights to follow up on the interaction. These ANOVAs revealed a significant main effect of phase in all three flights (first: *F*_2.04, 52.99_ = 9.45, *p* < .001, *η*_*p*_^*2*^ = .27; second: *F*_2.21, 57.40_ = 13.29, *p* < .001, *η*_*p*_^*2*^ = .34; third: *F*_3. 78_ = 3.96, *p* = .011, *η*_*p*_^*2*^ = .13). Further *t*-tests for the first flight showed that all pairs were significant (*p* < .05), except the pair (c1/t2: *p* = .533). In the second flight, all pairs reached significance (*p* < .05), except one pair that was marginally significant (t1/t2: *p* = .055). In the third flight, only 4 out of 6 pairs were significant (*p* < .05; c1/c2: *p* = .651, t1/t2: *p* = .393).

#### Skin conductance level (SCL)

**Fig. 5:**
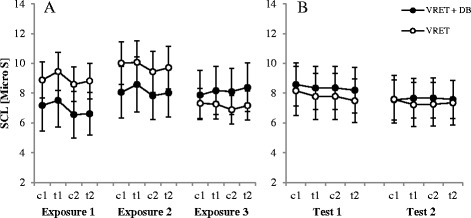
Skin conductance level (SCL) during the exposure (5**a**) and test sessions (5**b**). Note. *VRET* = Virtual reality exposure therapy, *DB* = diaphragmatic breathing, *c1/c2* = calm, *t1/t2* = turbulence. Graphs represent the mean of the SCL during the exposures (1–3) and the test sessions (1–2) for both groups. Standard errors are represented as error bars

As shown in Fig. 5, SCL remained relatively stable during the three VR exposure flights. ANOVA revealed significant main effects of phase (*F*_1.76, 47.47_ = 6.64, *p* = .004, *η*_*p*_^*2*^ = .20) No other effects were significant. A follow-up analysis of the three flights separately showed a significant main effect of phase for the first and second flights (first: *F*_1.34, 37.47_ = 4.85, *p* = .024, *η*_*p*_^*2*^ = .15; second: *F*_2.30, 64.27_ = 5.66, *p* = .004, *η*_*p*_^*2*^ = 0.17), but not for the third flight (*p* = .384). Follow up *t*-tests showed that in the first flight all pairs except two (c1/t2: *p* = .375, c2/t2: *p* = .503) were significant. Similarly, in the second flight we could see the same pattern (c1/t1: *p* = .155, c1/t2: *p* = .435). This indicates that the turbulence manipulation was still influential within the first two flights but not in the third flight.

Excluding the older participants, ANOVA revealed significant main effects of phase (*F*_1.76, 44. 06_ = 6.02, *p* = .007, *η*_*p*_^*2*^ = .19) No other effects were significant. A follow-up analysis of the three flights separately showed a significant main effect of phase for the first and second flight (first: *F*_1.31, 34.07_ = 5.13, *p* = .022, *η*_*p*_^*2*^ = .17; second: *F*_2.33, 60.66_ = 5.45, *p* = .005, *η*_*p*_^*2*^ = .17), but not for the third flight (*p* = .493).

Follow up *t*-tests showed that in the first flight all pairs except two (c1/t2: *p* = .329, c2/t2: *p* = .475) were significant. Similarly, in the second flight we could see the same pattern (c1/t1: *p* = .225, c1/t2: *p* = .282).

### Test flights in VR

#### Self-reported fear

The fear ratings changed within flights—depending on the flight phase—and between flights. These changes are indicated by significant main effects of flight (*F*_1.27_ = 45.29, *p* < .001, *η*_*p*_^*2*^ = .63) and phase (*F*_1.40, 37.90_ = 17.40, *p* < .001, *η*_*p*_^*2*^ = .39). Excluding the two participants aged > 55, there was an effect of flight (*F*_1,25_ = 45.90, *p* < .001, *η*_*p*_^*2*^ = .65) and phase (*F*_1.40, 34.99_ = 17.51, *p* < .001, *η*_*p*_^*2*^ = .41).

#### Heart rate (HR)

The HR decreased both within and between flights during the test flights. These patterns are partially reflected in the statistical analysis. The ANOVA revealed a significant main effect of flight (*F*_1, 27_ = 4.82, *p* = .037, *η*_*p*_^*2*^ = .15) and a Flight x Phase interaction (*F*_1.73, 46.59_ = 5.84, *p* = .008, *η*_*p*_^*2*^ = .18). To follow up on the interaction, we conducted two ANOVAs, one for each flight with phase as the within subject factor. Only the first flight phase was marginally significant (*F*_1.31, 36.54_ = 3.52, *p* = .058, *η*_*p*_^*2*^ = .11). The *t*-tests within the first phase showed a significant difference or marginally significant differences between t2 and c1 (*p* = .051), t1 (*p* = .038) and c2 (*p* = .068) and a marginally significant difference between t1 and c2 (*p* = .058).

Excluding the two older participants, the ANOVA revealed no significant main effect of flight (*p* = .074) but a Flight x Phase interaction (*F*_1.72, 43.01_ = 5.40, *p* = .011, *η*_*p*_^*2*^ = .18). To follow up on the interaction, we conducted two ANOVAs, one for each flight with phase as the within subject factor. Neither for the first (*p* = .079) nor for the second (*p* = .104) flight there was a significant effect of phase.

#### Skin conductance level (SCL)

The SCL remained relatively stable during the test flights. No significant differences were observed (a trend for the main effect flight was observed (*F*_1, 26_ = 3.04, *p* = .093, *η*_*p*_^*2*^ = .11)). Excluding the two participants older than 55 there were no significant differences.

### Relation between self-efficacy and fear of flying

Table 3 shows the correlations between self-efficacy (pre exposure, post test, and self-efficacy change, i.e. the difference in self-efficacy score between pre exposure and post test) with FFS pre exposure and post test levels and FFS reduction (pre exposure – Test). A significant negative correlation between self-efficacy post test and the FFS score post test was discovered (*r *= −.77, *p* < .001). However, there was no significant correlation between self-efficacy change and reduction in the FFS in either group.

**Table 3:** Correlation of self-efficacy with FFS

	FFS BL	FFS post	FFS reduction
VRET + breathing			
self-efficacy pre exposure	-.50	-.45	.08
self-efficacy post test	-.16	-.54*	.47
self-efficacy change	.55*	.18	-.26
VRET			
self-efficacy pre exposure	-.61*	-.35	-.25
self-efficacy post test	-.42	-.77**	.42
self-efficacy change	.14	-.34	-.52

Note. Correlation of self-efficacy (pre session 1, post session 2, and self-efficacy change) with FFS at baseline (BL), after the test session (post) and FFS reduction (BL – post) are given. *FFS* Fear of Flying Scale; **p < .01; *p < .05

Excluding two participants older than 55 years of age there was a significant negative correlation between self-efficacy post test and the FFS score post test both in the VRET group (*r* = −.73, *p* = .007) and in the VRET + breathing group (*r* = −.54, *p* = .039). Furthermore, there was a significant negative correlation between self-efficacy pre exposure and both the baseline FFS score (*r* = −.68, *p* = .015) and the FFS score post test (*r* = −.60, *p* = .038) in the VRET group. Furthermore, there was a significant correlation between the self-efficacy change and the FFS baseline score in the VRET + breathing group (*r* = .55, *p* = .033). Apart from that there were no significant correlations between self-efficacy (pre exposure, post test, and self-efficacy change) and the FFS scores (pre exposure and post test levels and FFS reduction).

### Manipulation check

#### Respiratory frequency

Descriptive analyses indicated that patients in the VRET + DB group were able to maintain the learned breathing frequency (four seconds inhale - six seconds exhale, six cycles per minute). Patients were instructed to practice diaphragmatic breathing between exposure and test sessions. According to their reports, patients practiced on average 4.93 times (*SD* = 2.29), with a mean practice time of 8.67 min (*SD* = 4.43). Importantly, even without verbal instruction, patients continued to keep their breathing rhythm at 6 cycles per minute (mean breath duration was 9.88 (*SD* = 0.91) and 10.35 (*SD* = 2.1) in the exposure and test sessions, respectively).

## Discussion

The present study investigated the effect of diaphragmatic breathing as an additional coping strategy during VRET in patients with aviophobia. A significant reduction of fear of flying was confirmed by the FFS measures in both groups. As predicted, this reduction in fear of flying was more evident in the VRET + DB group than in the VRET group, although at trend level only. These results support the hypothesis that actions that could be considered to represent distraction – in this case DB, a relaxation exercise – do not necessarily have a negative impact on treatment efficacy.

Our findings correspond with the results reported by Milosevic and Radomsky [34], who investigated the effects of safety equipment during exposure treatment for snake-fearful participants. The authors discovered that safety equipment did not reduce treatment effects. Similar results were reported by Johnstone and Page [11]: patients with spider-phobia who were engaged in a distracting conversation during treatment showed better treatment effects compared to patients that had to maintain a focused conversation during the exposure. Jones and Menzies [35] found that coping strategies could enhance self-efficacy.

As expected, VRET with and without DB resulted in a significant reduction of subjective fear ratings and HR during exposure therapy as well as during the second exposure session, which did not include a therapist’s guidance. It is noteworthy that subjective fear ratings and HR continued to decrease during the test session without a therapist’s guidance, which implies that after just one session of exposure treatment patients with aviophobia were able to benefit from the therapy. In the VRET + DB group, the patients showed a high level of compliance with the rhythm trained in session 1 without any additional auditory breathing instructions, which indicates that patients can learn diaphragmatic breathing in only one session.

Regarding psychophysiological process variables, we found HR decreased in all exposure trials, while SCL remained constant throughout most exposure trials, reacting only at the onset (flight 1) of turbulence. A possible explanation for this result could be the course of the exposure session with turbulences during the flight and accelerations and decelerations during start and landing, which might have impeded or overshadowed habituation. Similarly, in a study on virtual reality exposure to heights which also involved repeated stressors (looking down) during exposure there was no habituation of the SCL either [36]. SCL is sensitive to novelty and would probably only have decreased steadily if the flights during the exposure were calm, which was not the case in this study. However, the fact that during the third of the three flights, turbulences no longer increased SCL indicates a desensitization effect to turbulences. Both HR and fear ratings increased during turbulent flight phases, which corresponds with the findings of Trimmel, Burger, Langer, and Trimmel [37].

Bandura [38] showed that social cognition theory emphasizes self-efficacy with regards to coping with threatening situations as the key to therapeutic change. No significant differences in self-efficacy were evident in our study between the groups. This corresponds with the conclusions from Craske et al. [13], in that an increase in self-efficacy may not be the mechanism underlying the better response of the VRET + DB group to the treatment. Rather a combination of cognitive and physiological factors may account for the enhanced treatment effects in the VRET + DB group. With respect to cognitive processes, a possible underlying mechanism could be an increase in the perception of control: By attentively influencing their breathing, patients feel like they have control over their body, which reduces the feeling of helplessness usually experienced while anxious [39]. In following studies, the effect of perceived control over the situation while undergoing VRET should be investigated. As far as physiological factors are concerned, diaphragmatic breathing can lead to muscle relaxation, decreased arousal of the autonomic nervous system and a slower heart rate [15]. The reduced level of arousal might enhance extinction learning.

Some limitations to this study should be taken into account. First, there are different types of aviophobia [40], which we did not assess. Therefore, we did not test whether our VR therapy protocol is adequate for all types of aviophobia. Second, we only investigated a limited number of participants in this pilot study (*n* = 29) and therefore the power of our study is low^1^. Further studies have to confirm the results of our current study. This could possibly explain non-significant results. Concerning the calculations of initial fear activation, we did not collect baseline data for HR and SCL, but instead used the last two minutes before the landing of the third flight, assuming that physiological arousal had reached its baseline level again. Moreover, there was a difference of about 10 years in age between the two groups. However, we are not aware of any studies indicating a relationship between age and treatment effects for aviophobia. Moreover, our participants did not differ in treatment-relevant variables such as initial fear ratings. Another important limitation is the fact that participants’ avoidance and fear post-treatment was only assessed under virtual reality test conditions and not on actual airplanes. In order to generalize the observed treatment effects to real flying, a follow-up investigating treatment outcome with flights on actual airplanes would be necessary.

Finally, being a pilot study, the experiment was conducted with a limited number of participants. Nevertheless, our results indicated that DB may be a beneficial supplement for VR exposure therapy in aviophobics. Further research including larger samples will be needed in order to confirm this view.

## Conclusion

In conclusion, the present study provides additional evidence for the effectiveness of VR exposure therapy as a treatment for aviophobia. Importantly, our results indicate that using diaphragmatic breathing as an additional strategy to VR exposure could possibly lead to higher reductions in fear of flying compared to VR exposure alone. Further studies should analyze whether this effect is specific to aviophobia, or if it is encountered in other anxiety disorders (e.g., panic disorder) as well.

## Abbreviations

ANOVA: Analysis of variance
ASI: Anxiety sensitivity index
BL: Baseline
DB: Diaphragmatic breathing
DSM: Diagnostic and statistical manual of mental disorders
FFS: Fear of flying scale
FSB: Flying phobia screening questionnaire
HMD: Head mounted display
HR: Heart rate
M: Mean
RR: Respiration rate
SCID: Structured clinical interview for DSM-IV disorders
SCL: Skin conductance level
SD: Standard deviation
VR: Virtual reality
VRET: Virtual reality exposure therapy

## Footnotes

1 According to post hoc power analysis a total sample size of N = 36 (with power of 0.8 and the observed effect size for the interaction) would have resulted in a significant effect.
